# Advanced Imaging Techniques for Radiotherapy Planning of Gliomas

**DOI:** 10.3390/cancers13051063

**Published:** 2021-03-03

**Authors:** Antonella Castellano, Michele Bailo, Francesco Cicone, Luciano Carideo, Natale Quartuccio, Pietro Mortini, Andrea Falini, Giuseppe Lucio Cascini, Giuseppe Minniti

**Affiliations:** 1 Neuroradiology Unit, IRCCS Ospedale San Raffaele and Vita-Salute San Raffaele University, 20132 Milan, Italy; castellano.antonella@hsr.it (A.C.); falini.andrea@hsr.it (A.F.); 2 Department of Neurosurgery and Gamma Knife Radiosurgery, IRCCS Ospedale San Raffaele and Vita-Salute San Raffaele University, 20132 Milan, Italy; bailo.michele@hsr.it (M.B.); mortini.pietro@hsr.it (P.M.); 3 Department of Experimental and Clinical Medicine, “Magna Graecia” University of Catanzaro, and Nuclear Medicine Unit, University Hospital “Mater Domini”, 88100 Catanzaro, Italy; cascini@unicz.it; 4 National Cancer Institute, G. Pascale Foundation, 80131 Naples, Italy; luciano.carideo@istitutotumori.na.it; 5 A.R.N.A.S. Ospedale Civico Di Cristina Benfratelli, 90144 Palermo, Italy; natale.quartuccio@arnascivico.it; 6 Radiation Oncology Unit, Department of Medicine, Surgery and Neurosciences, University of Siena, Policlinico Le Scotte, 53100 Siena, Italy; giuseppe.minniti@unisi.it; 7 IRCCS Neuromed, 86077 Pozzilli (IS), Italy

**Keywords:** radiation treatment planning, glioma, advanced MRI, magnetic resonance spectroscopy, perfusion-weighted imaging, diffusion-weighted imaging, hypoxia, PET, amino acid radiopharmaceuticals, FET

## Abstract

**Simple Summary:**

Conventional magnetic resonance imaging (MRI) sequences have known limitations in target delineation for radiation treatment (RT) planning of cerebral gliomas. Advanced physiology-based MRI techniques and radionuclide imaging techniques, including positron emission tomography (PET) with amino acid radiopharmaceuticals, may increase the specificity for glioma tissue characterization. Our work aims to provide a comprehensive review of the advanced MRI and PET imaging modalities that can complement conventional MRI for RT planning of gliomas. A detailed overview of their basic principles and clinical results is given based on the most updated literature.

**Abstract:**

The accuracy of target delineation in radiation treatment (RT) planning of cerebral gliomas is crucial to achieve high tumor control, while minimizing treatment-related toxicity. Conventional magnetic resonance imaging (MRI), including contrast-enhanced T1-weighted and fluid-attenuated inversion recovery (FLAIR) sequences, represents the current standard imaging modality for target volume delineation of gliomas. However, conventional sequences have limited capability to discriminate treatment-related changes from viable tumors, owing to the low specificity of increased blood-brain barrier permeability and peritumoral edema. Advanced physiology-based MRI techniques, such as MR spectroscopy, diffusion MRI and perfusion MRI, have been developed for the biological characterization of gliomas and may circumvent these limitations, providing additional metabolic, structural, and hemodynamic information for treatment planning and monitoring. Radionuclide imaging techniques, such as positron emission tomography (PET) with amino acid radiopharmaceuticals, are also increasingly used in the workup of primary brain tumors, and their integration in RT planning is being evaluated in specialized centers. This review focuses on the basic principles and clinical results of advanced MRI and PET imaging techniques that have promise as a complement to RT planning of gliomas.

## 1. Introduction

The mainstay of treatment for patients with either low-grade or high-grade gliomas (HGG) is surgical resection followed by a combination of radio and chemotherapy [1,2]. In patients with glioblastoma (GBM), standard treatment consists of maximal surgical resection, radiation treatment (RT) (60 Gy in 2-Gy fractions), and concomitant and adjuvant chemotherapy with temozolomide [3]. In elderly patients, a hypofractionated RT schedule (40 Gy in 15 daily fractions of 2.67-Gy) showed equivalent overall survival (OS), but lower toxicity compared with standard RT [4,5]. Based on the CCTG CE.6/EORTC 26062-22061 phase III trial, hypofractionated RT associated with concomitant and adjuvant temozolomide has become the standard treatment modality for elderly patients with GBM [6]. However, despite aggressive management, approximately 90% of GBM recur locally within two years [1,7]. Postoperative RT at doses of 54–59.4 Gy in 1.8 Gy per fraction is the standard of care for adult grade 2 and 3 gliomas, followed by procarbazine, lomustine, vincristine (PCV chemotherapy), or temozolomide chemotherapy [2].

Modern RT techniques, including intensity-modulated radiotherapy (IMRT), stereotactic RT (SRT), and radiosurgery (SRS), allow better conformality of dose to the target with a subsequent decrease in treatment-related complications [8]. However, late neurocognitive dysfunctions, presenting as diminishing mental capacity for working memory, learning ability, executive function, and attention, remain a major concern for patients with glioma receiving high dose radiation to large brain-volume [9,10].

In this regard, an accurate delineation of tumor volumes and organs at risk (OARs) is critical to ensure maximum target dose and sparing of the surrounding normal brain structures to maintain high tumor control, while minimizing treatment-related toxicity. Most radiation treatment centers are equipped with dedicated computed tomography (CT) scanners that provide precise geometric information of anatomical structures, as well as electron density information for accurate dose calculation in treatment planning systems. Magnetic Resonance Imaging (MRI), using postoperative contrast-enhanced T1-weighted and T2-weighted sequences, has progressively replaced CT imaging because of its excellent soft-tissue contrast, high spatial resolution, and widespread availability. Based on these characteristics, MRI represents the current standard imaging modality for glioma target volume delineation; however, conventional imaging does not provide biological information, such as regional blood volume and microstructural architecture. Limitations of conventional MRI sequences include: (1) Limited capability in differentiating between treatment-related changes and disease progression in previously treated gliomas [11,12,13]; (2) non-specific increase in blood-brain barrier (BBB) permeability in contrast-enhanced T1-weighted sequences, which can reflect BBB disruption rather than truly assessing tumor vascularity; and (3) non-specificity of T2-weighted signal abnormality [14,15,16,17].

To overcome the aforementioned limitations, advanced physiology-based MRI techniques have been developed for biological characterization of brain gliomas, such as MR spectroscopy (MRS), diffusion MRI (dMRI), and perfusion MRI (PWI), providing relevant metabolic, structural, and hemodynamic information for treatment planning and monitoring [18,19,20]. Radionuclide imaging techniques, such as positron emission tomography (PET), are also being increasingly used in the workup of primary brain tumors, as they can provide important diagnostic information regarding the delineation of tumor extent for treatment planning, for the diagnosis of treatment-related changes, and the assessment of treatment response [21,22,23,24].

In this article, we provide a comprehensive overview of the basic principles and clinical results of the advanced MRI and PET imaging modalities that can complement conventional MRI for RT planning of gliomas.

## 2. Standard Target Delineation for Gliomas

MRI, using pre- and postcontrast T1-weighted and T2-weighted sequences, typically fluid-attenuated inversion recovery (FLAIR) sequences, is mandatory for precise radiation treatment of gliomas [25]. For patients with GBM who underwent surgical resection, the RT dose is typically 60 Gy delivered in 2-Gy fractions. According to the European Organization for Research and Treatment of Cancer (EORTC) target delineation guidelines, the gross tumor volume (GTV) is the resection cavity plus any residual enhancing tumor as seen on the postoperative MRI. For patients who underwent surgical biopsy, the GTV is defined as the T1-weighted contrast-enhancing lesion. The clinical tumor volume (CTV), which includes areas of potential suspected microscopic tumor infiltration and possible paths of microscopic spread, is generated by adding a variable margin of 15–20 mm to the GTV constrained at anatomical borders, e.g., tentorium, falx cerebri, and bone [25]. The US Radiotherapy and Oncology Group (RTOG) recommends an alternative “two phases” target delineation. The initial GTV volume, which includes the surgical resection cavity/any residual enhancing tumor/surrounding edema plus a margin of 2 cm to generate the CTV, receives 46 Gy followed by a “boost” of additional 14 Gy to a smaller volume constituted by surgical resection cavity/any residual enhancing tumor plus a margin of 2 cm [7,25]. For both approaches, the planning target volume (PTV), which considers uncertainties of planning and setup, is generated by adding an isotropic margin of 3-5 mm from the CTV, based on the positioning and verification system used.

For grade 3 glioma, the typical total dose is 59.4 Gy at 1.8 Gy per fraction [26]. The GTV is represented by the lesion visible on post-contrast T1-weighted MRI sequences with 2–3 mm slice thickness or on FLAIR sequences in nonenhancing tumors. The CTV is usually generated by applying a margin of 10–20 mm anatomically constrained. For grade 2 glioma, where a dose of 50.4–54 Gy is employed in 28–30 fractions of 1.8 Gy per fraction, the GTV is the resection cavity plus any residual hyperintense tumor as seen on the postoperative T2/FLAIR sequences, and the CTV is the GTV plus an additional margin of 10–20 mm anatomically constrained. Finally, the PTV is generated by applying a margin of 3–5 mm.

However, it should be noted that the use of variable GTV-to-CTV margins of 10–20 mm represents an oversimplification of the disease biology and recurrence risk pattern [27]. A large CTV, which includes possible pathology not visible on standard MRI imaging, has the potential to better fit all microscopic tumor infiltration; however, this approach may increase the risk of neurocognitive decline and worsen the quality of life [28]. Moreover, conventional MRI techniques have clear limitations in the evaluation of glioma heterogeneity among different regions of the same tumor, such as metabolic changes and proliferative activity, hypoxia, and neovascularization/angiogenesis, which can, at least in part, explain their resistance to RT, and that can be specifically investigated using advanced MRI and PET imaging [23,29,30,31,32,33].

For recurrent gliomas undergoing reirradiation, the GTV is still defined as the visible lesion on MRI contrast-enhanced T1-weighted sequences; however, limited total doses and small GTV-to-CTV margins of 0-5 mm are usually utilized during reirradiation, either SRS or fractionated SRT, to limit the higher risk of radiation-induced brain necrosis associated with a second course of RT [34]. In this setting, using physiological and metabolic imaging may have a great impact on optimal target delineation.

## 3. Advanced Physiological MRI for RT Planning of Gliomas: Technical Background and Clinical Results

### 3.1. MR Spectroscopy (MRS)

Proton MR spectroscopy (^1^H-MRS) has been largely used to detect and quantify several endogenous cellular metabolites in brain gliomas in vivo, including choline (Cho), N-acetyl-aspartate (NAA), creatine (Cr), lactate (Lac), and lipid (Lip) [35]. As glioma malignancy and grade increase, an abnormal elevation of Cho is observed, due to the elevated membrane phospholipid turnover in actively proliferating tumors, whereas a decrease of the peaks of NAA, a marker of neuronal integrity, and Cr, a marker of bioenergy storage, is detected [36,37,38]. HGG often show the appearance of Lac and Lip peaks, byproducts of anaerobic glycolysis and tissue hypoxia, and cell death and necrosis [38]. As the absolute quantification of MRS-detected metabolites is technically challenging in a clinical setting, semi-quantitative assessment using metabolite ratios is often used. Furthermore, by combining MRS acquisition with spatial localization techniques similar to those used in generating anatomical MR images, a larger volume can be selected to acquire signals from multiple voxels across a 2D slice or 3D cubic volume, to generate semi-quantitative maps of the variations in levels of the different metabolites [39]. This method is known as MR spectroscopic imaging (MRSI), and despite the presence of significant challenges for robust acquisitions of good-quality data, it is the technique of choice for the integration in RT treatment planning. With modern MR systems in a specialized center, the acquisition time for clinically-adapted 3D whole-brain MRSI is on the order of 5–10 min, and the spatial resolution of the voxels obtained is typically 0.5–1 cm^3^ [40].

Correlation of in vivo MRSI parameters with ex vivo histologic features from image-guided tissue samples in patients with gliomas showed that areas of elevated Cho and reduced NAA relative to normal brain accurately correlate with regions of increased cellular proliferation, thus indicating a metabolically active part of the tumor [36,41]. The ratio of choline to NAA (Cho/NAA index or CNI), normalized to the contralateral normal tissue, has been used as a semi-quantitative metric to define the extension of the metabolic abnormality in GBM [36,42,43].

Furthermore, in studies comparing differences in the contrast-enhancing (CE) lesion, T2/FLAIR, and metabolic lesions for grade 3 glioma and GBM, the volume of Cho/NAA abnormality is often substantially larger than the contrast-enhancing region and sometimes extends beyond the margins of FLAIR abnormality, thus possibly detecting disease infiltration and predicting areas of newly enhanced lesions after chemoradiation therapy [44,45,46,47,48]. Therefore, in the context of glioma RT planning, MRSI may help improve microscopic disease coverage and prevent marginally recurrent disease [35,49,50,51].

There is interest in targeting MRSI metabolic abnormality by selectively escalating the dose using either IMRT ‘dose painting’ or SRS boosts. Dose painting approaches deliver spatially non-uniform doses with very steep dose gradients, improving normal tissue sparing. By using this method, it is possible to create highly heterogeneous dose distributions within a brain tumor, minimizing the doses to surrounding healthy tissues [52].

Ken et al. simulated the integration of MRSI in the treatment planning system (TPS) for GBM dose painting to prove the dosimetric feasibility of a simultaneous integrated boost (SIB) up to 72 Gy to the volume defined by a Cho/NAA ratio > 2 [53]. In a cohort of 16 GBM patients, they simulated three types of dosimetry plans, one conventional plan of 60-Gy in 3D conformational radiotherapy (3D-CRT), one 60-Gy plan in IMRT, and one 72-Gy plan in SIB-IMRT. Dosimetry plans of 72-Gy SIB-IMRT and 60-Gy IMRT showed a significantly decreased maximum dose to the brainstem (44.00 and 44.30 vs. 57.01 Gy) and decreased high-dose volumes to the normal brain compared to 60-Gy 3D-CRT (*p* < 0.05). This demonstrated that delivering standard doses to conventional targets and higher doses to new target volumes based on the areas of highest Cho/NAA abnormality is possible without increasing the dose to organs at risk [53].

A prospective phase II trial (trial NCT00253448) on 35 GBM patients incorporated a single 15–24 Gy Gamma Knife SRS boost in addition to standard chemoradiation, to be directed at high-risk disease defined by a Cho/NAA ratio >2. Acceptable toxicity and favorable OS compared with historical controls were reported. Specifically, the median survivals for recursive partitioning analysis (RPA) Class 4, 5, and 6 patients were 18.7, 12.5, and 3.9 months, respectively, compared with Radiation Therapy Oncology Group (RTOG) RT-alone historical control survivals of 11.1, 8.9, and 4.6 months. For the 16 of 35 patients who received concurrent TMZ in addition to protocol RT treatment, the median survival was 20.8 months, which compared favorably with the European Organization for Research and Treatment of Cancer (EORTC) historical controls of 14.6 months using RT and TMZ [54].

More recently, a multicenter prospective phase III trial in newly diagnosed GBM (SPECTRO GLIO, trial NCT01507506, estimated study completion date: March 2023) is evaluating the potential survival benefit of a simultaneous integrated boost of IMRT (72Gy/2.4Gy) delivered to the portion of the disease identified by a Cho/NAA > 2 at MRSI [55].

The feasibility of using MRSI to guide dose-escalated RT for newly diagnosed GBM is also under investigation in a single-arm multi-institutional trial using 3D MRSI (trial NCT03137888, estimated study completion in July 2021). The study utilizes the Brain Imaging Collaboration Suite (BrICS), a cloud platform developed by Gurbani et al. that integrates MRSI with standard MRI and enables team members from multiple institutions to work together in RT target delineation. Further outcomes of the study are the 1-year PFS, the OS, and the performance on neurocognitive and quality-of-life (QOL). Data from 18 patients treated using targets created in BrICS have been reported so far without severe toxicities [56].

Further investigation is needed to clarify the potential of MRSI in radiation treatment planning and to standardize the analysis of MRSI spectra [51,56]. In particular, the absence of a common platform across different vendors for 3D MRSI processing and the technical complexity of integrating spectral images in the treatment planning system still represent limitations to the wide implementation of this technique in the RT workflow.

Future directions will also include the possibility to non-invasively detect tumor-specific intracellular metabolites by MRSI, which may also have the potential to assist in treatment planning and monitoring. In particular, intratumoral accumulation of 2-hydroxyglutarate (2HG) resulting from the isocitrate dehydrogenase (IDH) gene mutation in brain gliomas can be quantified in vivo by MRSI [57,58]. 2HG has been recently used as a biomarker to detect the presence and spatial distribution of IDH-mutated cells in gliomas, and the tumor volume identified by 2HG MRSI extends beyond FLAIR pathologic volume in a significant number of patients with IDH-mutant gliomas [59], thus having important implications for radiotherapy planning of this molecular subtype of gliomas. Further studies are warranted to enhance the implementation of this method, still technically challenging, in the clinical setting.

### 3.2. Diffusion MR Imaging

Diffusion MR imaging (dMRI) allows probing molecular water diffusion within tissues, thus providing microscopic details about the architecture and cellularity of both normal and diseased tissues [60]. dMRI enables calculation of apparent diffusion coefficient (ADC), which quantifies the mean diffusivity of water molecules within each voxel (mm^2^/s) and contributes to estimate tumor hypercellularity and grade in brain gliomas non-invasively, as largely reported in the literature [61,62]. An inverse correlation between ADC and glioma cell proliferation has been largely reported, with minimum ADC values reflecting the areas of highest cellularity, thus correlating with malignancy and predicting survival [62,63]. An alternative approach analyzes the low segment of the ADC histogram of the entire tumor volume, which has been shown to carry prognostic information in both newly-diagnosed and recurrent GBM treated with antiangiogenic therapy [64,65]. However, no prospective studies have proven any advantage of integrating ADC in the RT planning of newly diagnosed GBM in terms of survival or time-to-progression. Furthermore, the interest in using this modality was tempered after a recent report of the SPECTRO GLIO trial suggested that neither ADC nor perfusion-derived relative cerebral blood volume (rCBV, see below) has distinct specificity in predicting recurrence on a per voxel basis [66]. However, a major caveat of this trial was the use of dMRI with a ‘standard’ degree of diffusion-weighting of the sequence (namely, a b-value of 1000 s/mm^2^). More recently, it has been shown that by increasing the sensitivity to smaller diffusion movements, i.e., by increasing the b-value, dMRI can reveal proliferating tumor tissue with highly restricted diffusivity, thus increasing specificity [67]. As such, high b-value dMRI (up to 3000–4000 s/mm^2^) may allow the identification of hypercellular subvolumes of GBM that predict PFS and may extend into or beyond the FLAIR abnormality, thus falling outside of the typical high-dose RT volume [67]. The possibility to use these hypercellular subvolumes identified by high b-value dMRI as a boost target for RT could be tested in future studies.

Interestingly, voxelwise ADC changes during RT have been used to investigate and quantify physiological and pathological variations of tumor cellularity, which could provide information for individual patient adaptation of RT [68,69]. The early appearance of a large number of tumor voxels with increased ADC during RT is associated with a better response [68].

Technical challenges of using diffusion images and ADC maps to define a boost, or adaptive target include the typical geometric distortions and susceptibility to B0-field inhomogeneity of the echo-planar (EPI) pulse sequences used for dMRI, that can be partly overcome using multi-shot EPI, high parallel imaging factors and the newest acquisition designs using multiple phase-encoding directions for dMRI and blip-up/blip-down EPI distortion correction methods [70].

#### Diffusion Tensor Imaging (DTI) and MR Tractography

Diffusion tensor imaging (DTI) is a dMRI technique that quantifies the amount and orientation of hindered water diffusion within tissues [60]. As water diffusion is anisotropic in brain white matter, reflecting its organization in bundles of fibers running in parallel, DTI can also be used to map the underlying tissue microstructure [60]. The DTI-derived fractional anisotropy (FA) map reflects fiber directionality and density, as well as the axonal diameter and white matter myelination [71]. DTI has been exploited to depict the spatial orientation of the white matter fiber tracts in the brain by a method called fiber tracking or MR tractography [72]. MR tractography is the only non-invasive method that enables to identify in vivo the main fiber tracts adjacent to or inside brain tumors [73] and is commonly used in glioma preoperative setting to improve neurosurgical planning, guiding the surgical approach to prevent damages to relevant tracts [74]. The rationale for using DTI and MR tractography in RT target delineation is the histopathological evidence that invasive glioma cells migrate preferentially along white matter fiber tracts [75,76]. Furthermore, mathematical models of glioma growth are typically improved by incorporating DTI anisotropy for simulation [77]. Consequently, DTI abnormalities have been largely employed to define the extent of peritumoral microinfiltration beyond the apparent borders on conventional MR imaging [78], supported by histopathological validation from DTI-guided brain biopsies [79]. In addition, the pattern of DTI abnormalities has been shown to predict patterns of tumor recurrence in HGG [80], and the location of progressive tumor spread [81]. As the peritumoral white matter abnormality depicted by DTI can be used to predict the trajectory of invasive tumor cells, then this information could be used to inform RT treatment planning [27,82,83]. This can be particularly relevant for diffusely infiltrating lower-grade gliomas or GBMs featuring large non-contrast-enhancing tumor portions, whose extension might be better characterized by DTI signatures. An anisotropic expansion that considers DTI abnormality may maximize the chances of treating migrating cancer cells, while minimizing the amount of brain tissue exposed to high doses of ionizing radiation [27]. It is worth noting that, in the setting of RT planning, target volumes defined by DTI-derived mathematical glioma growth models showed scarce overlap with the standard CTV, possibly related to the different information conveyed by this technique [84].

Jena et al. retrospectively compared standard planning techniques with individualized plans based on DTI in seven patients with biopsy-proven HGG by performing a dosimetry study to prove that DTI could be used as the basis of a dose escalation strategy [83]. The volume of DTI-based abnormality was added to the conventional GTV to encompass areas at high risk of tumor involvement, and then patient-based, individualized CTVs (CTVI) were generated by adding a 1 cm margin to the DTI+GTV volume. In all cases, DTI was shown to reduce PTV size (mean 35%, range 18–46%), resulting in escalated doses (mean 67 Gy, range 64–74 Gy), with normal tissue complication probability (NTCP) levels that matched the conventional treatment plans. The authors concluded that DTI could individualize RT target volumes by excluding areas of the unaffected brain from the target volume, with consequent CTV reduction. The use of a non-uniform margin from GTV to CTV would allow significant dose escalation, while restricting the risk of normal tissue damage to acceptable levels [83].

Berberat et al. evaluated the feasibility of using DTI for RT target volume delineation in 13 GBM patients [82]. A DTI-CTV was generated by adding the DTI abnormality to the contrast-enhancing lesion, and this volume was isotropically expanded by 1 cm and then extended for an additional 1 cm in length and width along the visible, apparently normal, white matter tracts adjacent to the tumor to create the DTI-PTV. DTI-CTV was smaller when compared to a conventional T2-weighted CTV (*p* < 0.005), thus suggesting that DTI-CTV may detect more specifically tumor invasion rather than tumor plus peritumoral edema, as for T2-weighted CTV. Compared to the conventional PTV, the DTI-PTV showed a trend towards volume reduction. It is worth noting that, although these DTI-based volumes were smaller than conventional volumes, they still included sites of tumor recurrence. As such, the extension of CTV along the abnormal tensor tracts preserves coverage of glioma cells’ routes of spreading whilst sparing uninvolved brain, which seems a promising approach to individualize RT planning for GBM patients [82].

In light of these feasibility studies, a multicenter, prospective longitudinal observational cohort study in patients with GBM is ongoing (PRaM-GBM, trial NCT03294434, estimated study completion by June 2021) to establish a DTI-based model that could accurately predict where GBM will progress after treatment, therefore evaluating its utility to optimize radiation treatment planning. In this study, a comparison of amino-acid PET and perfusion-derived rCBV with DTI biomarker is also planned.

More recently, Jordan et al. proposed an innovative approach to combine MR tractography in RT planning by developing a tool for translating a tractography dataset into a white matter path length (WMPL) map. In this map, each voxel’s quantitative value represents the minimum distance (in mm) between the voxel and the GTV along white matter pathways [27]. These WMPL maps can be loaded into an RT planning software to modify the treatment volume anisotropically. The method was tested in a retrospective cohort of 13 GBM patients, three of whom had marginal recurrences using a standard isotropic technique. Using WMPL to define target volumes, two of three marginal recurrences would have been included in the target volume, and all other recurrences would have remained within the target volume, with a median target volume 19% smaller than the isotropic technique. This proof-of-concept work lays the groundwork for future studies to evaluate the clinical value of incorporating tractography modeling into treatment planning [27].

The integration of MR tractography has also been proposed in the context of inverse planning, by defining a target dose to the tumor tissue and dose-volume constraints to relevant white matter tracts, and then determining, via an optimization process, the treatment plan which best matches all the input criteria. Wang et al. used DTI and functional MRI cortical activations (BOLD-fMRI) to localize the motor corticospinal tracts (CSTs) and primary motor cortices in a retrospective cohort of 20 patients with HGG [85]. For each patient, three different treatment plans were considered: A three-dimensional conformal radiation treatment (3DCRT) plan and an IMRT plan, both considering the standard morphological organs at risk (OARs), as well an IMRT plan which also included CSTs and primary motor cortices (PMCs) among the OARs. The authors found that the maximum and mean dose (Dmax and Dmean) to the ipsilateral and contralateral PMC and CST regions were considerably decreased in the IMRT plans, including tractography and fMRI data, possibly reducing the probability of late radiation injury to these structures [85]. Similar results of dose-reduction to relevant white matter tracts located near the RT target volumes were described in another study from Igaki et al., which incorporated the CST as OAR in the IMRT plan of GBM and comparing the dose sparing with respect to conventional IMRT [86].

**Figure 1:**
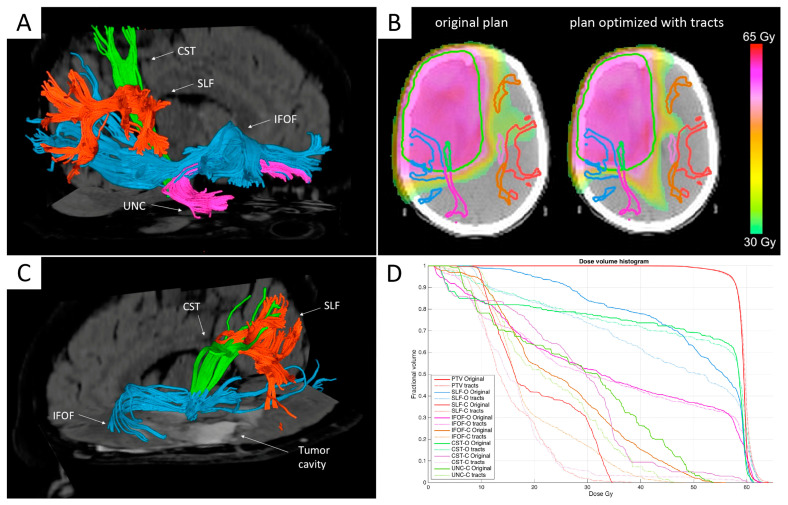
Integration of multiple white matter tracts as depicted by MR Tractography in the tomotherapy RT planning. (**A**,**C**) Post-surgical MR tractography analysis of a glioblastoma (GBM) patient: The upper image (**A**) shows the reconstructed tracts in the contralateral (healthy) hemisphere, while the lower image (**C**) shows the tracts surrounding the surgical cavity in the ipsilateral (affected) hemisphere. CST = corticospinal tract; IFOF = Inferior fronto-occipital fasciculus; UNC = uncinate fasciculus; SLF = superior longitudinal fasciculus. (**B**) Comparison of dose distributions for the original plan (on the left) and the tract-optimized plan (on the right), showing the different dose conformation outside the target and the preservation of relevant tracts. (**D**). The dose-volume histograms (DVH) data for the two planning modalities in B is shown. DVH for each tract in the original plan is represented with solid lines, while DVH for each tract in the new plan incorporating the fibers is represented with dotted lines. ‘I’ indicates ipsilateral tracts, while ‘C’ indicates contralateral tracts. A significant reduction of the dose delivered to tracts was observed when fibers were included in the optimization process, which was more relevant for contralateral tracts. No significant differences were found in PTV coverage between the original plans and the optimized plans incorporating fiber tracts (solid and dotted red lines). Adapted with permission from [87].

More recently, Altabella et al. evaluated the feasibility of integrating multiple white matter tracts as depicted by MR tractography in the tomotherapy RT planning in a retrospective dosimetric study of 19 HGG patients [87]. The authors evaluated three intra-hemispheric associative fiber bundles involved in language or visuospatial attention networks (superior longitudinal fascicle, inferior fronto-occipital fascicle, and uncinate fascicle) and the projection motor fibers of the CST (Figure 1). For all patients, the original plans without tracts were compared with the optimized plans incorporating the fibers, the latter demonstrating a significant Dmean and Dmax reduction for most of the tracts, with more relevant dose sparing for contralateral tracts (*p* < 0.0001) and without significant differences in terms of PTV [87]. Future studies are warranted to assess the clinical benefits of MR tractography-guided dose sparing on long-term cognitive dysfunctions and the impact of this approach on patients’ neurological outcomes and quality of life.

### 3.3. Perfusion MRI

Perfusion-weighted imaging (PWI) studies tissue microcirculation by measuring blood flow and vascular permeability, thus quantifying changes associated with neoangiogenesis in brain gliomas [20]. The dynamic susceptibility contrast (DSC) PWI technique is the most commonly used perfusion method, based on susceptibility variations during the first pass of a gadolinium-based contrast agent bolus through the tumor microvasculature [88]. From the resulting T2*-weighted dynamic data, cerebral blood volume maps relative to normal brain (rCBV) can be derived [88]. rCBV strongly correlates with glioma microvessel density at histopathology [89] and is the most validated perfusion parameter to predict tumor grade and malignancy [90], as well as survival outcomes in patients with brain gliomas [91,92]. In a retrospective study with 189 patients with gliomas, elevated mean rCBV values were significantly associated with a shorter time to progression, independent of histopathological grade [91].

In addition, increased rCBV values have been reported to extend beyond regions of T1-contrast enhancement with high frequency (up to 50%), suggesting a possible role for PWI in identifying tumor invasion and assisting with RT planning [93]. However, as mentioned earlier in this review, a recent report of the SPECTRO GLIO trial (NCT01507506) suggested that rCBV does not add specificity to the prediction of GBM sites of relapse [66]. In the setting of newly diagnosed gliomas, using rCBV alone to guide RT dose painting has been reported only anecdotally [94]. Some evidence on the utility of DSC-PWI in RT target delineation has been reported in the re-irradiation of HGG [95], and is discussed later in a specific paragraph of this review.

Other PWI techniques, such as dynamic contrast-enhanced (DCE) MRI, can provide a quantitative evaluation of vascular permeability and plasma volume by assessing changes in T1 tissue relaxivity over 5–10 min after the injection of the gadolinium-based contrast agent. The resulting images are analyzed with a pharmacodynamic model to provide maps of biologically relevant parameters, such as the contrast transfer constant (K^trans^), the fractional volume of the extravascular-extracellular space (ve), and the fractional volume of the intravascular compartment (vp) [96]. The extent and severity of vascular leakage have been shown to correlate with tumor aggressiveness and outcome in HGG [97]. High vascular leakage of glioma tissue, quantified by DCE-derived K^trans^, is a significant predictor of progression-free survival [98]. More recently, Nguyen et al. explored the prognostic role of DCE-MRI in a prospective study of 46 patients with newly diagnosed gliomas, showing that high preoperative K^trans^ obtained from DCE-MRI is associated with poorer outcome in patients with newly diagnosed low- and high-grade gliomas [99]. Other DCE-derived parameters, such as the plasma volume (vp), demonstrated a similar accuracy when compared to DSC-derived rCBV in estimating microvessel density and preoperative grading, having the advantage of a higher spatial resolution of DCE and insensitivity to susceptibility-induced geometric distortion typical of DSC acquisitions [100]. Nonetheless, to date, there are no published data about using DCE-derived parameters alone for RT planning of gliomas.

It is worth noting that integrating PWI-derived parameters in the RT workflow requires standardization of PWI methods within and across sites, which is strongly advocated to ensure their reproducibility and reliability in clinical practice [100]. Automated software tools with sufficient validation are highly desirable, being aware that PWI threshold values strongly depend on scan parameters and post-processing methods.

### 3.4. Multiparametric MR-Guided RT Planning

**Figure 2:**
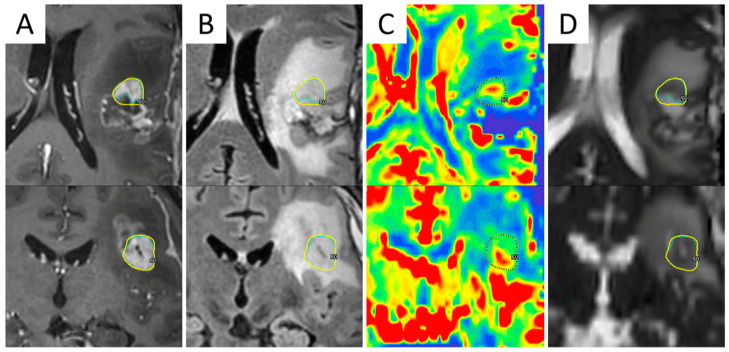
Integration of advanced MRI sequences in a Gamma Knife radiosurgical planning of a 44-year-old male patient with recurrent WHO grade III astrocytoma after multimodal first-line therapy and recent redo surgery with maximum safe resection. Axial (upper row) and coronal (bottom row) views of contrast-enhanced T1-weighted (**A**) and fluid-attenuated inversion recovery (FLAIR) (**B**) MRI images, co-registered with relative cerebral blood volume (rCBV) map (**C**) and apparent diffusion coefficient (ADC) map (**D**), demonstrating how different sequences identify different volumes of disease. The final target was determined as the nodule showing hyper-intensity in post-contrast T1-weighted sequences, hypo-intensity in ADC maps, and elevated perfusion values on the rCBV map.

The combination of multiple advanced MRI techniques may enhance their implementation in the RT planning setting (Figure 2). Their integration with dosimetry may help identify voxels at risk for progression and allow voxel-level risk-adapted dose escalation to subclinical disease while sparing normal tissue [101].

Wahl et al. have shown that a combination of PWI DCE-MRI and high b-value dMRI into a multiparametric imaging signature predicts PFS and shows spatial correspondence with patterns of failure. In particular, they found that pre-therapy sub-volumes with overlapping hypercellularity and increased CBV derived from DCE-MRI will progress in nearly all cases [102]. Based on these findings, Kim et al. launched a phase II study (trial NCT02805179, estimated study completion date February 2021) to evaluate the feasibility and efficacy of a multiparametric assessment of tumor hyperperfusion and hypercellularity. This study combines DCE-MRI and high b-value dMRI to guide intensified (75 Gy/30 fractions) RT on areas at highest risk of treatment failure in patients with newly diagnosed glioblastoma [103]. A preliminary report described the workflow and the initial imaging outcomes of the first 12 patients. On average, the combined hypercellular/hypervascular volumes were 1.8 times smaller than the contrast-enhancing abnormality and 10 times smaller than the FLAIR abnormality. In particular, hypercellular volume and high tumor perfusion volume identified largely distinct regions and showed 57% overlap with the enhancing abnormality, with minimal-to-no extension outside the FLAIR. The feasibility of implementing a workflow for multiparametric MR-guided RT against prognostically distinct tumor subregions that differ substantially from the T1-Gd-enhancing high-risk boost volumes was demonstrated, although survival outcomes are still awaited from this study [103].

Furthermore, automatic segmentation methods integrating advanced multimodal MRI have been recently proposed to reduce the intrinsic intra- and inter-observer variability of target delineation and to tackle the intrinsic complexity of data analysis [104,105]. Guo et al. recently proposed a fusion method for auto-segmentation of gliomas in RT planning using multiparametric dMRI derived ADC, DTI-derived FA, and DSC-derived rCBV. The method was preliminarily applied on four clinical image datasets (two low-grade and two high-grade astrocytomas). When compared with manually delineated GTVs, high accuracy and efficiency of the automatic segmentation methods were achieved, suggesting the potential of utilizing functional multiparametric images for GTV definition in precision RT planning of gliomas [105]. The limitations of multimodality approaches for target delineation are related to the blurred tumor border on advanced MRI maps, due to the relatively low resolution with respect to conventional MRI, as well as to the partial volume effects and the inherent noise in image acquisition that could produce a negative influence on the segmentation results [104,105].

## 4. PET Radiopharmaceuticals for RT Planning of Gliomas: Physiology and Clinical Results

### 4.1. [^18^F]Fluorodeoxyglucose

[^18^F]Fluorodeoxyglucose (FDG) is the workhorse of PET. Its intracellular transport is primarily mediated by the sodium-independent glucose transporter 1 (GLUT-1), which is constitutively expressed by the endothelial cells forming the BBB and by the glial cells [106]. Owing to the physiologically high brain FDG uptake, microscopic disease beyond MRI-visible abnormalities is unlikely to be detected by FDG PET. In line with this, previous studies on RT planning of gliomas have demonstrated that the tumor portion identified by FDG PET is generally smaller than that identified by standard MRI [107,108]. Escalating radiation doses to FDG-positive tumor portions did not show a survival advantage [109].

### 4.2. Amino Acid Analogs

Amino acid PET tracers, namely, [^11^C]methyl-l-methionine (MET), O-(2-[^18^F]fluoroethyl)-l-tyrosine (FET) and 3,4-dihydroxy-6-[^18^F]fluoro-l-phenylalanine (F-DOPA), are now being successfully used in the management of patients with primary or secondary brain tumors [23,110,111]. In contrast to FDG, they are characterized by high tumor-to-background ratios (TBR), making them the radiopharmaceuticals of choice in brain tumor imaging. The cellular uptake of radiolabeled amino acids is based on the expression of the sodium-independent large neutral amino acid transporters LAT1 and LAT2 on tumor cells [112]. This mechanism is independent of BBB permeability, enabling radiolabeled amino acids to depict non-contrast-enhancing tumor portions, including during anti-angiogenic therapy [65,113,114]. On the other hand, unspecific uptake may occur at sites of BBB disruption and inflammation [115]. Additionally, there are recent reports showing that concomitant therapies with dexamethasone or temozolomide have an impact on amino acid uptake of normal-appearing brain structures [116,117,118]. These variables should be carefully evaluated in light of potential effects on target delineation, particularly in the recurrent setting.

With the exception of MET, amino acid radiopharmaceuticals are not incorporated into proteins. Despite this and other biological differences, including the metabolism of F-DOPA by the aromatic amino acid decarboxylase in dopaminergic and serotoninergic neurons, no significant differences in terms of tumor detection have been shown between the three most widely available radiolabeled amino acids [119,120,121].

**Figure 3:**
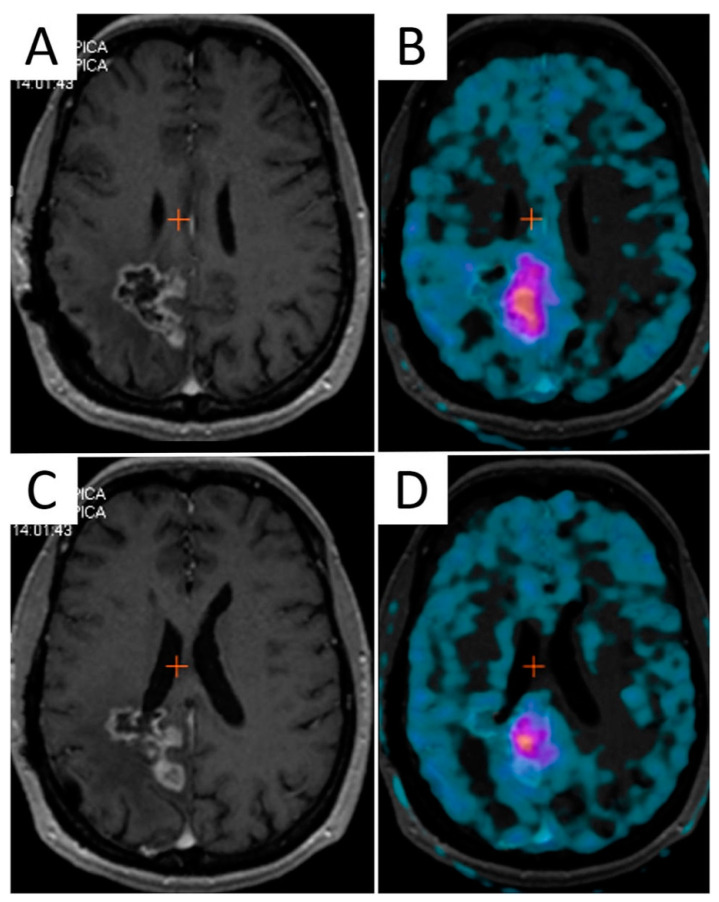
The mismatch between contrast-enhanced T1-weighted MRI and 3,4-dihydroxy-6-[^18^F]fluoro-L-phenylalanine (F-DOPA) PET/CT in a 45-year-old female patient with recurrent GBM after multimodal first-line therapy. Two axial contrast-enhanced T1-weighted images (**A**,**C**) along with corresponding F-DOPA PET/CT slices (**B**,**D**) identifying different volumes of disease.

Amino acid PET has shown better accuracy than standard MRI in the definition of brain tumor extent [122], and its inclusion in the surgical planning demonstrated a positive impact on survival [123]. Additionally, the biological tumor volume (BTV) identified by amino acid PET demonstrated a significant prognostic value either after surgery [124] or after postoperative chemo/radiotherapy in GBM. These findings provide a strong rationale for the incorporation of amino acid PET in RT planning [125] (Figure 3).

### 4.3. MET

MET was first used for brain tumor imaging about three decades ago and has long been considered the standard PET radiopharmaceutical in this setting. Nevertheless, because of the short half-life of Carbon-11 (20 min), MET cannot be commercialized and is available only to centers having an on-site cyclotron. Several original studies have analyzed the role of MET in radiotherapy planning. As early as 2005, Grosu AL et al. showed that the BTV defined by MET-PET was substantially different from that defined using traditional radiological investigations (MRI or CT) in 39 patients with HGG following surgery. MET tumor uptake, defined by a TBR of 1.7, extended non-uniformly beyond Gd enhancement on MRI in 74% of cases and was identified outside the hyperintensity areas on T2-weighted MRI in 50% of patients. Of note, in 28% of patients, amino acid uptake extended >25 mm beyond the MRI abnormalities seen on contrast-enhanced T1-weighted sequences. In contrast, Gd enhancement and T2 hyperintensities extended outside the MET uptake in 69% and 100% of cases, respectively [126].

These findings have significant implications for target volumes delineation and for sparing normal brain tissue from unnecessary radiation. A later study of Matsuo M et al. in 32 patients with newly diagnosed GBM following surgery showed that a margin of at least 20 mm in T2-weighted sequences is necessary to cover MET PET signal with good accuracy [127]. In this paper, a TBR of 1.3 was chosen as the threshold for malignancy based on the results of a former biopsy-controlled study [128].

Navarria et al. investigated the role of integrated MET and MRI for RT planning in 69 patients with HGG following surgical resection [129]. They found that, in all patients, the BTV defined on MET-PET was included within the CTV based on FLAIR sequences. In contrast, the MET-PET signal was outside the CTV based on contrast-enhanced T1-weighted images in 50% of cases. This suggests that the use of MET would not change the target volumes if these are defined based on FLAIR sequences; nevertheless, the integration of MET could significantly modify the CTV in case this is defined on contrast-enhanced T1-weighted sequences. However, the criteria for the definition of tumor extent on MET PET were not specified in this study [129]. Iuchi et al. designed a study to establish the correlation between postoperative MET uptake and relapse patterns in 22 patients affected by GBM. They established dose thresholds needed to control regions with different tumor-to-background indices on MET. A region with MET TBR < 2.12 might be controlled by a total dose of 60 Gy using standard fractionation (2 Gy/fraction), while a region with MET uptake index < 1.41 might be controlled by a standard total dose of 40 Gy. These results suggest that the optimal radiation dose for tumor control could be determined based on MET uptake in individual cases [130]. The pattern of failure after radiochemotherapy was retrospectively correlated with MET PET, not used for RT planning, by Lee et al. in 26 patients with newly diagnosed GBM. MET-BTV was defined by a threshold of 1.5 times the mean cerebellar uptake. The analysis showed that non-central failures are more common in patients with suboptimal coverage of MET BTV [131].

### 4.4. [^18^F]FET

The results obtained by Grosu et al. with MET [126] were confirmed by Weber and co-workers in 19 patients with HGG imaged by FET. In this study, the BTV identified by FET using 40% of maximum standardized uptake value (SUVmax) as a threshold for tumor definition, extended more than 20 mm beyond Gd-enhanced GTV in 32% of cases. BTV and GTV were substantially discordant in the majority of patients (95%) [132]. A subsequent analysis of the relapse patterns in 10 patients from the same cohort showed a similar recurrent tumor volume outside either the GTV or the BTV [133]. Using a different threshold for tumor volume definition (i.e., TBR > 1.5) in 17 patients with GBM, other authors concluded that FET-based BTV was significantly larger than MRI-based GTV [134]. Rieken et al. showed that MRI-based PTV would not cover the FET-based GTV in 17% of 41 patients at first irradiation or at recurrence, suggesting that amino acid PET should be routinely included in the RT planning [135]. In line with this, a more recent study showed that the CTV (GTV + 20 mm) based on Gd-enhancement would cover about 90% of the FET-based GTV. In this study, it is speculated that patients with GBM would benefit most from integrating FET in RT planning, as they have larger FET-positive volumes of disease compared to grade III gliomas [136]. Potential candidates to undergo FET-aided RT planning might be particularly those patients with GBMs featuring non-contrast-enhancing tumor portions, which eventually turn out to be FET-positive [137].

Harat et al. prospectively evaluated the integration of dual-time point FET PET, acquired prior to primary radiochemotherapy, on the RT treatment planning and prediction of recurrence of 34 patients with GBM. They showed that PET-based GTV extended beyond GTV plus 20 mm margins in 26.5% cases. Furthermore, the recurrence pattern analysis showed that progressions occurred most often in the FET-GTV than in the MRI-GTV (70% vs. 57% of cases) [138]. These results are in accordance with those of Lundemann et al., who analyzed 50 patients with GBM showing that the overlap between treatment volumes and the recurrent tumor is highest for RT planning integrating contrast-enhanced MRI and PET compared to RT planning based on either modality alone [139]. The pattern of recurrence was also analyzed retrospectively by Fleischmann et al. in 36 patients with GBM who underwent FET PET before primary radiochemotherapy. They showed that integrating FET would reduce the GTV expansion from 20 mm to 15 mm compared to RT planning based on contrast-enhanced MRI alone [140]. These latter studies used the same TBR threshold of 1.6 for tumor contouring on PET [135,136,137,138,139,140]. Finally, a dose escalation strategy based on a FET-adapted RT boost has been evaluated prospectively with no apparent clinical benefit [141]. Nevertheless, a follow-up study of GBM relapse patterns after FET-adapted radiotherapy showed that a CTV based on FET + 7 mm margin would cover 100% of relapses, while significantly reducing the PTV [142].

### 4.5. F-DOPA

F-DOPA PET has been more rarely used for RT planning of brain gliomas so far. Kosztyla et al. compared target delineation using MRI and F-DOPA PET in 19 patients with newly diagnosed HGG. They found that PET-based volumes were significantly larger than MRI-based volumes. Nevertheless, all but one documented recurrence extended beyond the PET GTV, and most were contained by a 20 mm margin on the MRI GTV. Therefore, they concluded that it is unclear whether treatment planning using F-DOPA PET would yield better treatment outcomes. A major limitation of this work was that the criteria for PET positivity were not specified [143]. In a later study, the same authors investigated the feasibility of using F-DOPA PET for dose painting with volumetric modulated arc therapy (VMAT) in 10 patients with HGG. F-DOPA PET could achieve dose-escalated coverage to BTV without increasing the dose to cranial OARs, suggesting that this approach would offer better disease control than conventional RT for HGG [144].

Potential advantages and current limitations of advanced MRI techniques and amino acid PET for first-line RT planning of glioma have been summarized in Table 1

## 5. Imaging of Hypoxia

### 5.1. Hypoxia-Targeting Radiopharmaceutical: [^18^F]FMISO, [^18^F]FAZA, [^64^Cu]Cu-ATSM

Tumor hypoxia is characterized by an oxygen concentration below critical O_2_ levels and triggers several molecular, biological, and clinical effects, making it a negative prognostic marker in nearly all solid tumors, including GBM. A relevant feature of tumor hypoxia is the lower cellular radiosensitivity, requiring a higher dose to achieve equivalent biological effects under normoxic conditions [145]. Several PET radiopharmaceuticals have been developed to target hypoxia, including nitroimidazole-based and non-nitroimidazole compounds [146,147]. The most representative radiopharmaceuticals of the first class are 3-[^18^F]fluoro-1-(2-nitro-1-imidazolyl)-2-propanol (FMISO) and [^18^F]fluoroazomycin arabinoside (FAZA) [148]. Nitroimidazole analogs penetrate the cell by passive diffusion, and under low oxygen concentration (pO_2_ < 10 mmHg), are progressively reduced, leading to the production of reactive radicals that bind covalently and irreversibly, to intracellular molecules. Conversely, in the presence of molecular oxygen, these tracers are oxidized back and exit the cell. An additional mechanism of tracer accumulation might be related to glutathione conjugation [149]. Compared to FMISO, FAZA shows faster clearance and superior image contrast [150]. Amongst non-nitroimidazole radiopharmaceuticals, the complex of Cu(II) with diacetyl-bis(N^4^-methylthiosemicarbazone) (ATSM) was studied in more detail [151]. Cu-ATSM enters into the cells by passive diffusion. Then, copper (II) is reduced to copper (I) in the mitochondria. Cu(I)-ATSM remains trapped in hypoxic cells, due to its unstable nature, leading to the progressive dissociation of the complex in H_2_-ATSM and free Cu(I). By contrast, in normoxic cells, Cu(I)-ATSM is reoxidized and exits the cells. ^64^Cu-ATSM presents potential advantages for RT planning, including its half-life (12.7 h) and higher TBR than nitroimidazole-based tracers [146,147]. However, the retention mechanism of Cu-ATSM has been questioned, as it also accumulates in non-hypoxic cells depending on cellular redox metabolism [152,153].

**Figure 4:**
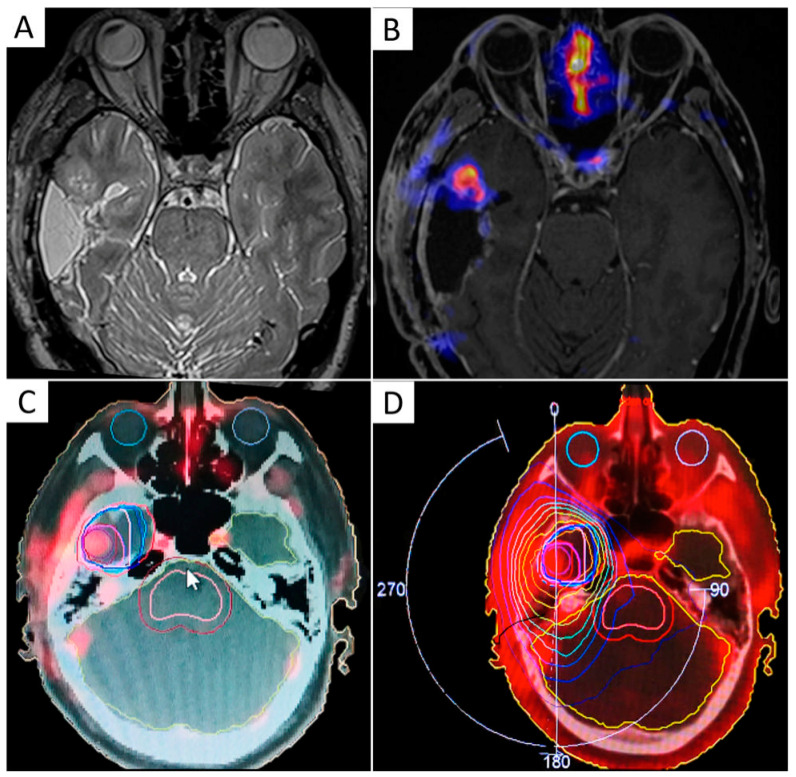
Example of a volumetric modulated arc therapy (VMAT) sequential boost of radiation to hypoxia-positive recurrent GBM. T2-weighted and contrast-enhanced T1-weighted MRI sequences were acquired along with ^64^Cu-diacetyl-bis(N^4^-methylthiosemicarbazone) (ATSM) PET on a hybrid 3T PET/MR scanner, three hours after radiopharmaceutical injection (**A**,**B**). 37.5 Gy were delivered in 15 daily fractions to the surgical cavity followed by a boost of radiation (5 Gy) to the ^64^Cu-ATSM-positive tumor region, indicating chronic hypoxia (**C**,**D**).

It has been shown that Hypoxia PET imaging may aid RT planning in patients with lung and head and neck cancer by safely guiding dose escalation to tumor regions with higher tracer uptake [154,155,156]. Uptake of hypoxia-targeting radiopharmaceuticals in brain tumors was found to correlate with the expression of endogenous markers of hypoxia, such as HIF-1α [157,158], and demonstrated a negative impact on survival outcomes [159,160,161]. Hypoxia PET has been used preclinically to guide radiation treatment in a rat GBM model [162]. However, only a few clinical experiences are available on patients with HGG undergoing hypoxia PET scans before and after RT, and a real PET-based treatment plan is missing in these cases [163,164] (Figure 4). Narita et al. documented reoxygenation in two patients with GBM undergoing FMISO PET scan before and after RT plus temozolomide, suggesting that the hypoxia is not spatially fixed over time and sequential hypoxia PET scans may be necessary for adaptive RT [164].

In summary, several hypoxia-targeting radiopharmaceuticals are available for clinical use and have shown prognostic and therapeutic relevance for solid tumors. Nevertheless, the application of hypoxia-PET in RT planning of glioma has been limited so far. Well-designed trials should evaluate whether hypoxia PET-based RT planning is feasible and advantageous for patients with glioma.

### 5.2. MRI Markers for Hypoxia

There is evidence that MRI techniques may contribute to hypoxia imaging, in addition to radionuclide techniques [165,166]. The main advantages of MRI biomarkers are the higher spatial and temporal resolution compared to PET biomarkers (about 3–4 mm), the repeatability (over hours and days), and the use of a non-ionizing technique. In addition, PET tracers are delivered to the hypoxic tumor cells via the bloodstream. However, GBM vascularization is highly perturbed, which could affect the radiopharmaceutical biodistribution, particularly in anoxic areas without functional vascularization. Radiopharmaceutical delivery may not be achieved in these regions, resulting in low tracer availability. Vessel permeability may also have an impact on radiopharmaceutical biodistribution if more hydrophilic tracers are used [166].

Quantitative Blood-oxygen-level-dependent (qBOLD) MR imaging exploits the intrinsic paramagnetic properties of deoxy-hemoglobin to infer data regarding blood oxygenation [167]. This approach estimates the number of quantitative metrics, including oxygen-extraction fraction (OEF) and cerebral metabolic rate of oxygen (CMRO_2_). Low-grade gliomas exhibit higher OEF and lower CMRO_2_, while HGG showed lower OEF, higher CMRO_2_, and higher neovascularization markers [168]. This has been interpreted in light of the additional oxygen-demand (CMRO_2_) of HGG, that drives neo-angiogenesis, supplying oxygen without an OEF, due to the abnormal and inefficient neoangiogenetic network [168]. In two recent studies, Stadlbauer et al. have combined the qBOLD-derived oxygen metabolism biomarkers with quantitative metrics of neovascularization derived from DSC-PWI in two cohorts of newly diagnosed GBM to depict and quantify the heterogeneity of tumor microenvironments (TMEs) [169,170]. By using this multiparametric approach, the authors identified non-invasively six main clusters or TMEs in GBM: Necrosis, hypoxia with defective neovasculature, hypoxia with functional neovasculature, normoxia with functional neovasculature, glycolysis without neovasculature, glycolysis with functional neovasculature [169]. By quantifying the different TME volume fractions, two different metabolic phenotypes for newly diagnosed GBM were uncovered, reflecting the dominating metabolic strategy for energy production: A glycolytic phenotype with stable, functional neovasculature, and a necrotic/hypoxic phenotype with a high proportion of unstable defective dysfunctional neovasculature and more aggressive tumor behavior. The glycolytic phenotype showed longer PFS [169], and tended to switch to the necrotic/hypoxic phenotype at recurrence, with a significantly higher rate of multifocality of the recurrent lesions [170]. If confirmed in larger studies, these data indicate qBOLD MRI as a valuable method for non-invasive characterization of hypoxic TME in a clinical setting, and possibly, in RT planning.

## 6. Target Delineation in the Re-Treatment Setting

**Figure 5:**
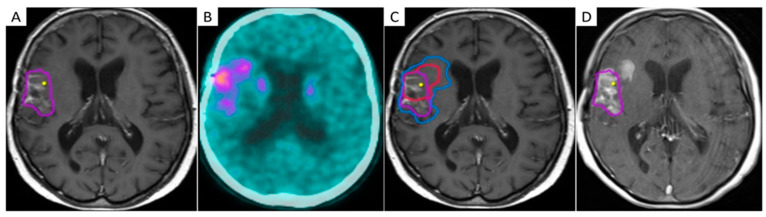
Integration of F-DOPA PET/CT in a LINAC-based radiosurgical planning of a 55-year-old female patient with recurrent GBM. (**A**) shows GTV based on contrast-enhanced T1-weighted MRI images; (**B**) shows remarkable F-DOPA uptake extending beyond contrast-enhancement. The final target volume was delineated by taking into account the F-DOPA-positive signal (**C**). Three-month follow-up (**D**) shows tumor progression at the site of pre-RT increased F-DOPA uptake, despite salvage irradiation.

In recurrent gliomas, standard procedures have not yet been established. However, the aim is to limit the re-irradiated volume to the absolutely necessary, i.e., to the active tumor region, to enhance efficacy and decrease possible neurotoxicity. The delineation of tumor volume should, therefore, be restricted to the contrast-enhancing T1 abnormality. Unfortunately, anatomical contrast-enhanced T1-weighted MRI is not suited for differentiating tumor progression from radiation necrosis and therapy-related changes [95,171,172]. For these reasons, imaging techniques capable of better detailing the entire extent of tumor recurrence and patterns of infiltration are needed to optimize radiation treatment volumes [51] (Figure 5).

### 6.1. Advanced MRI: MRS, dMRI, and PWI

MRSI-derived Cho/NAA abnormality may be relevant for defining the target volume for Gamma Knife radiosurgery (GK-SRS) or other re-irradiation forms in recurrent gliomas. Early retrospective studies demonstrated that patients with recurrent GBM showing areas of elevated Cho/NAA ratio outside of the target volume for GK-SRS had worse OS survival than patients for whom the Cho/NAA abnormality was fully treated [173,174]. In a retrospective evaluation of 26 patients with recurrent GBM treated with GK-SRS, Chuang et al. reported that metabolic disease as measured by Cho/NAA ratio > 2 extends beyond the contrast-enhancing lesion in the majority of cases. For some patients, the incorporation of this metabolically active tissue would make the radiosurgical target volume too large to be treated with such focal irradiation. However, this MRSI-based assessment would contraindicate an RT treatment that is likely to be ineffective, potentially allowing early adoption of alternative strategies [175]. Furthermore, serial follow-up with MRSI in the same patients showed a decrease in Cho, stable Cr, and increased NAA indicative of response to GK-SRS in the majority of patients, thus improving the interpretation of tumor-enhancement changes and the close monitoring of treatment response [175].

As for dMRI integration in the re-irradiation setting, additional RT dose painting or boost target based on ADC lowest values has been proposed by Orlandi et al. in a simulation study in recurrent GBM [52]. The feasibility of dose painting guided by the ADC signal for the re-irradiation of five recurrent GBM patients was tested, demonstrating a satisfactory degree of accuracy of the deliverable plans. Boost techniques should reasonably be performed in the first part of the treatment either as an additional or a simultaneous boost technique to impact more effectively on tumor cell proliferation, infiltration, migration, and neovascularization, which, together with hypoxia, are known to be the main causes of radio-resistance [52]. Conversely, if the boost is introduced during or at the end (sequential boost) of a Stupp therapy, the ADC signal could be potentially modified by the already received radiation dose [67], and the delivery time may be unfavorable, due to the presence of a more active tumor repopulation [52].

PWI has been proposed in the re-irradiation setting based on of early studies showing that quantitative DSC-derived metric of rCBV correlates with histological tumor fraction and OS in recurrent GBM [176]. Wang et al. explored the treatment effect of quantitative DSC-PWI-guided GK-SRS as salvage treatment in a prospective study on a cohort of 26 consecutive patients with recurrent HGG [95]. The GTV was defined as the high perfusion area on absolute CBV maps [177]. The median PFS after GKRS was eight months, which is more favorable than that obtained in prior studies that did not use absolute CBV maps for planning [178,179]. Conversely, no obvious treatment benefit in terms of OS was demonstrated using the quantitative PWI method proposed in this study. A small number of patients (3 out of 26) suffered from mild to moderate radiotoxicity, as the high perfusion area was larger than the contrast-enhancing area [95]. These results suggested that quantitative DSC-PWI-guided GKRS is feasible for treating recurrent HGG, even if these outcomes still deserve validation in larger cohorts of patients and randomized trials.

### 6.2. Amino Acid Radiopharmaceuticals

Amino acid radiopharmaceuticals have also been used in the re-irradiation setting. Grosu et al. included 44 patients with HGG (33 GBM, 9 anaplastic gliomas, and 2 gliosarcomas) in a study using MET-PET or ^123^I-methyl-tyrosine for RT planning [180]. Treatment planning, based on PET(SPECT)/CT/MRI imaging (PTV = GTV + 3 mm expansion), was associated with improved survival in comparison to treatment planning using CT/MRI alone (median survival time 9 vs. 5 months, respectively, *p* = 0.03) [180]. Miwa et al. also described the impact of MET-PET for reirradiation purposes with IMRT in 21 patients affected by GBM [181]. Following Kracht et al. [128], they used a TBR threshold of 1.3 for defining MET-PET positivity. GTV was defined in consensus, based on PET/CT/MRI image fusion, and the PTV was generated using a 3 mm isotropic expansion. Survival rates and toxicity profiles appeared favorable compared to similar previous works (OS rate from reirradiation = 11 months, with 6-month and 1-year OS rates = 71.4% and 38.1%, respectively), suggesting that the use of both MET-PET/CT/MRI image fusion and hypofractionated SRT might lead to an improved therapeutic ratio, especially in pre-treated patients with HGG [181].

More recently, Møller et al. performed a Phase I dose-volume escalation trial integrating FET-based BTV (>1.6 TBR threshold) into the RT planning of 31 patients with recurrent HGG [182]. The PTV was defined by a 2 mm expansion of the combination between FET BTV and contrast-enhanced MRI GTV. They found that both FET-based BTV and MRI-GTV (cystic/necrotic cavities subtracted) were independent OS prognosticators. However, no objective responses were identified, and survival outcomes were very poor, possibly due to the heavy pre-treatment of this patient population. The addition of a 7 Gy RT boost on the FET-positive BTV did not show any survival advantage, and overall, the toxicities observed were not negligible [183]. The authors suggested that FET PET may be used to support the decision of avoiding re-treatment in patients who will unlikely benefit from it, owing to a too large tumor burden [182]. The results of a recent multimodality imaging study on 41 patients with recurrent GBM were more promising. RT was planned based either on contrast-enhanced T1-weighted sequences or on FET PET (TBR >1.7–1.8), or on a combination thereof (CTV = GTV + 3 mm; PTV = CTV + 1–2 mm margin). The recurrence pattern analysis suggested that recurrence sites were more closely related to FET-based than MRI-based GTV, though the difference did not reach statistical significance [184]. A fully hybrid PET/MR study was conducted on seven patients with HGG. RT planning was based on contrast-enhanced MRI GTV plus 10 mm margins, adjusted to include the FET positive volume (TBR >1.6). The pattern of recurrence analysis indicated that combining the information of PET and MR may reduce the safety margin from 10 to 3 mm [185]. Lastly, the impact of amino acid PET on target delineation of GBM at recurrence is being evaluated by a prospective phase II trial (trial NCT01252459) whose results are still awaited [186].

Potential advantages and current limitations of advanced MRI techniques and amino acid PET in the re-irradiation setting of glioma have been summarized in Table 2.

## 7. Combination of Advanced MRI and PET

Several studies compared the information provided by amino acid PET and MRSI [32,187], dMRI [188,189,190,191], PWI [192,193,194,195,196,197,198] or combinations thereof [199,200,201,202,203], either acquired simultaneously on dedicated PET/MR scanners or co-registered after performing acquisition and processing on independent machines. These studies, already reviewed elsewhere [21,22,204], most often reported a poor spatial agreement between advanced multimodality MRI and PET techniques. Therefore, it can be concluded that these advanced techniques could complement each other. Nevertheless, their added value over conventional imaging should be evaluated in different specific clinical settings as it may vary depending on the relevant question to be answered.

So far, there are very few available studies directly comparing advanced MRI techniques with amino acid PET in the setting of RT planning. Dissaux et al. compared FET PET with standard MRI sequences and DSC PWI in 30 patients with newly diagnosed HGG scheduled for concomitant chemoradiotherapy. Overlap volumes and spatial similarity between contrast-enhanced MRI-based GTV and FET-based GTV were low, with more than 5 mL of FET-based GTV falling outside the MRI-based CTV (GTV + 20 mm). In contrast, there was a larger agreement between MRI-based GTV and rCBV tumor volume. The authors concluded that MRI underestimates the metabolically active tumor volume and that FET PET is expected to improve the accuracy of target volume delineation, although follow-up clinical information and patterns of recurrence were not provided [205].

Another study, mentioned earlier in this review, compared dMRI with contrast-enhanced MRI and FET PET for RT planning of GBM at recurrence [184]. The tumor volume encompassing areas of restricted diffusion (GTV-ADC_low_) showed poor overlap with PET-based (median non-overlapping volume = 69.5%) or MRI-based GTV (median non-overlapping volume = 72.2%). The recurrent pattern analysis showed a significantly smaller overlap between recurrent tumor volume and GTV-ADC_low_ compared with PET-based or conventional MRI-based GTV [184].

## 8. Conclusions

Advanced physiology-based MRI techniques and amino acid PET demonstrate higher specificity with respect to conventional MRI for characterizing the biological attitude of cerebral gliomas and are being increasingly used in RT planning in specialized centers and in prospective single- and multi-institutional trials. Amongst the advanced MRI techniques, MRS allows the most accurate depiction of tumor metabolism, thus appearing as a promising imaging method for RT planning integration, especially by using MRSI. However, its widespread application outside specialized centers is still limited by significant technical challenges. On the other hand, dMRI has been proven to be a sensitive, but indirect, method for characterization of glioma tissue microstructure and cellularity, easy to be implemented on clinical scanners. DTI and MR tractography may be useful complements in RT planning as they can help define the anisotropic tumor expansion and invasion of normal white matter structures. The information provided by PWI is relevant for characterization and quantification of tumor neoangiogenesis, but notwithstanding its undoubtful value in predicting glioma prognosis and monitoring of treatments, its impact on RT planning deserves confirmation in larger prospective studies and requires standardization of PWI methods within and across sites. Taking advantage of the wide availability of MRI and its ability to obtain different biological information within a single exam, the combination of multiple advanced MRI techniques may enhance their implementation in the RT planning setting. Amino acid PET is an extremely robust imaging technique that depicts the actual tumor extent with better accuracy than conventional MRI. The inclusion of amino acid PET in RT planning can change the planned treatment volumes in a significant number of patients, and the most recent studies showed a promising correlation between PET-positive tumor volume and patterns of relapse. Larger, prospective studies are warranted to correlate the changes of RT targets according to the information derived from PET and MRI with biological and molecular tumor changes, and to assess the possible clinical impact on treatment efficacy.

## Figures and Tables

**Table 1:** Summary of findings on advanced imaging techniques for first-line RT planning of gliomas.

First Line RT Treatment					
Advanced Imaging Modality	RT Planning Technique	Retrospective/ Simulation Studies Available	Prospective Studies Available	Potential Advantages	Limitations
MRSI	Dose escalation and GTV expansion based on increased Cho/NAA ratio	YES	YES	Reduced marginal and in-field recurrence, improved survival outcomes, reduced toxicity	Technically demanding
dMRI (ADC)	Dose escalation and GTV expansion on regions with reduced ADC (hypercellularity)	NO	NO	Better definition of hypercellular subvolumes identified by high b-value dMRI	EPI distortions may hamper image registration to define a boost or adaptive target
DTI	Anisotropic PTV expansion based on DTI abnormality (peritumoral microinfiltration); Dose painting	YES	NO	Better planning conformation according to tumor infiltrating pattern: Reduced toxicity and reduced out-of-field recurrences	Limited data available on survival benefit
MR Tractography	Inverse planning using eloquent tracts as OAR	YES	NO	Reduced toxicity, improved quality of life	No data on the impact on long-term cognitive dysfunction
PWI	Dose escalation and GTV expansion on regions with increased rCBV	NO	NO	Better definition of hypervascular areas; better tumor coverage	Lack of standardization of PWI acquisition and analysis; no data available on survival benefit
Amino acid PET ^1^	Inclusion of PET-BTV in RT planning	YES	YES	Better tumor coverage; better tumor control	Modification of RT planning depends on the PET segmentation method; limited data on survival benefit

^1^ Different amino acid radiopharmaceuticals are considered equivalent. NAA, N-acetyl-aspartate; Cho, choline; DTI, diffusion tensor imaging; OAR, organs at risk; PWI, perfusion MRI; BTV, biological tumor volume.

**Table 2:** Summary of findings of advanced imaging techniques in the re-irradiation setting of gliomas.

Re-Irradiation Setting					
Advanced Imaging Modality	RT Planning Technique	Retrospective/ Simulation Studies Available	Prospective Studies Available	Potential Advantages	Limitations
MRSI	Inclusion of regions with increased Cho/NAA ratio	YES	NO	Patient selection based on expected tumor coverage	Treatment volumes too large, probably unfeasible
dMRI (ADC)	Dose painting or simultaneous integrated boost based on reduced ADC (hypercellularity)	YES	NO	Better tumor coverage; better tumor control	Limited data available
PWI	Target delineation according to high rCBV regions	NO	YES	Improved survival outcomes in preliminary series	Lack of standardization of PWI acquisition and analysis; larger PTV, increased toxicity
Amino acid PET ^1^	Inclusion of PET-BTV in RT planning	YES	YES	Better tumor coverage; improved survival outcomes	Modification of RT planning depends on the PET segmentation method; survival benefit still unproven

^1^ Different amino acid radiopharmaceuticals are considered equivalent.
